# Cellular and molecular mechanisms of repair in acute and chronic wound healing

**DOI:** 10.1111/bjd.13954

**Published:** 2015-07-14

**Authors:** P Martin, R Nunan

**Affiliations:** 1 Schools of Biochemistry and Physiology & Pharmacology, University of BristolBristol, U.K; 2 School of Medicine, University of CardiffCardiff, U.K

## Abstract

**Summary:**

A considerable understanding of the fundamental cellular and molecular mechanisms underpinning healthy acute wound healing has been gleaned from studying various animal models, and we are now unravelling the mechanisms that lead to chronic wounds and pathological healing including fibrosis. A small cut will normally heal in days through tight orchestration of cell migration and appropriate levels of inflammation, innervation and angiogenesis. Major surgeries may take several weeks to heal and leave behind a noticeable scar. At the extreme end, chronic wounds – defined as a barrier defect that has not healed in 3 months – have become a major therapeutic challenge throughout the Western world and will only increase as our populations advance in age, and with the increasing incidence of diabetes, obesity and vascular disorders. Here we describe the clinical problems and how, through better dialogue between basic researchers and clinicians, we may extend our current knowledge to enable the development of novel potential therapeutic treatments.

**What's already known about this topic?:**

1. Much is known about the sequence of events contributing to normal healing.
2. The two pathologies of wound healing are chronic wounds and scarring.

**What does this study add?:**

1. We explain how the cell and molecular mechanisms of healing guide the therapeutic strategies.
2. We introduce zebrafish and the fruit fly, *Drosophila* as novel wound healing models.
3. We highlight unanswered questions and future directions for wound healing research.

Wound healing after damage to the skin involves a complex interplay between many cellular players of the skin, primarily keratinocytes, fibroblasts, endothelial cells of vessels and recruited immune cells, and their associated extracellular matrix (Fig.1). In healthy individuals, restoration of a functional epidermal barrier is highly efficient, whereas repair of the deeper dermal layer is less perfect and results in scar formation with a substantial loss of original tissue structure and function. When the normal repair response goes awry there are two major outcomes: either an ulcerative skin defect (chronic wound) or excessive formation of scar tissue (hypertrophic scar or keloid).

**Figure 1:**
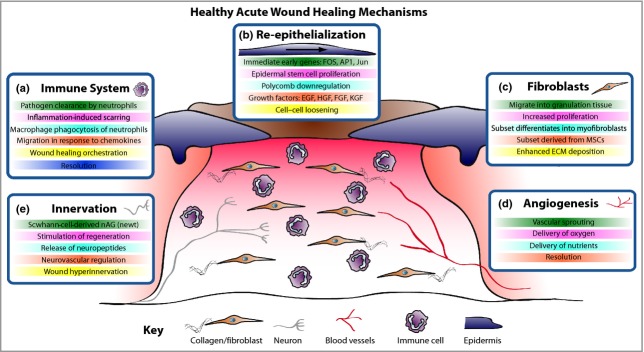
Acute wound healing mechanisms. The healing of an acute wound involves coordinated cellular and molecular responses. (a) Initially immune cells migrate to the wound site and, in addition to clearing invading pathogens, in part they also orchestrate the healing process. (b) Cut epidermal edges upregulate wound-associated genes, thus enabling collective cell migration. (c) Local and blood-borne fibroblasts proliferate and migrate to form the wound granulation tissue, provide structure and signalling cues and deposit new extracellular matrix (ECM). Some fibroblasts differentiate into myofibroblasts to aid wound contraction. (d) The wound bed is perfused with oxygen and nutrients through new blood vessels derived by angiogenesis. (e) Wound healing rates exhibit a positive correlation with innervation, but hyperinnervation after wound closure could contribute to neuropathic pain. EGF, epidermal growth factor; HGF, hepatocyte growth factor; FGF, fibroblast growth factor; KGF, keratinocyte growth factor; MSC, mesenchymal stem cell; nAG, newt anterior gradient protein.

Tissue repair is a universal phenomenon across all multicellular organisms, and so we presume that many conserved mechanisms can be analysed in models more experimentally tractable than humans, and subsequently extrapolated back to the clinic for potential therapeutic benefit. Because of similarities to human skin, pig models of wound healing were initially used for investigating repair mechanisms,^1^ and remain a popular model for preclinical trials of potential therapeutics. However, cost issues and genetic opportunities have seen rodents take over as the predominant models for investigating the fundamental cellular and molecular mechanisms underlying tissue repair.

Much of what we know about the cellular and genetic players in wound healing and the relative time courses of the various phases of skin repair come from studies in mice. Transgenic and knockout mouse studies have provided opportunities to investigate the functions of many genes that turn out to have key roles during skin healing.^2^ The recent advent of Cre-lox and other tissue-specific and conditional knockout approaches has enabled more thorough investigations than earlier studies with whole-body knockouts.

## The basic cell biology of wound re-epithelialization

The wound epithelium repairs both from cut wound edges and also from the stumps of hair and sweat gland appendages. Mouse wound transcriptome studies have revealed numerous genes upregulated after damage, and many of these gene inductions occur in the wound edge epithelium, extending back up to 70 or more rows of cells from the cut wound edge.^3^–^5^ The earliest gene upregulations are classic immediate early genes, including *Ap1*, *Fos* and *Jun*,^6^ and the krox zinc finger transcription factors.^7^ These presumably function as part of the transcriptional activation machinery for the several hundred genes that are subsequently upregulated in these cells, and enable a surge in cell proliferation and associated epidermal migration of a leading tongue of keratinocytes at the interface between the scab and healthy wound granulation tissue. It has become clear that at least some of these late-activated genes, for example epidermal growth factor receptor, are generally kept silent by histone methylation marks deposited by the polycomb family of epigenetic regulators, but the polycombs are downregulated and these marks are removed soon after wounding so that the silenced genes can now be ‘open for business’ and available for transcriptional activation.^8^

Before migrating forward, wound-edge keratinocytes must change their cell-to-matrix adhesions. These adhesions will previously have bonded basal epidermal cells to the basement membrane, but during the repair process they must facilitate migration over a new wound-specific, fibrin-rich matrix. Several integrins are switched off in order for cells to detach from the basement membrane, and others now become essential for wound migration. For example, keratinocyte-specific knockout of β1-integrins in mice leads to severe retardation in wound re-epithelialization.^9^ Cell–cell junctions must also be modified. A recent study showed how the desmosomal junctions linking the advancing wound keratinocytes become ‘looser’ and calcium dependent; this switch is likely to be protein kinase C alpha dependent, because PKCα^−/−^ mice fail to change these adhesions and exhibit delayed healing.^10^ Proteases, in particular several matrix metalloproteases (MMPs), are needed to clip the links between integrins and collagen as the epidermis advances over the wound substratum.^11^

Re-epithelialization is then activated by several driving growth factors, including hepatocyte growth factor (HGF) and one or more members of the fibroblast growth factor (FGF) and epidermal growth factor (EGF) families. Studies have shown that keratinocyte-specific knockout of c-met (HGF receptor) or FGF receptors 1 and 2 leads to severe retardation of the wound epidermis,^12^,^13^ and a global knockout of the EGF receptor led to a lag in re-epithelialization in mice.^14^

Generally, epidermal cells are lost following any skin injury and they must be replaced by cell proliferation, which occurs largely in an epithelial zone back from the migrating epidermal tongue.^15^ The contribution to new keratinocytes by stem cells and the source of such stem cells are not entirely clear; labelled cells from the stem cell-dense bulge region of adjacent hair follicles move up and, at least initially, can populate the denuded territory.^16^ A more permanent source of new keratinocytes appears to be the non-bulge-region follicular cells that make similar-sized contributions to those of cells derived from stem cells outside of the hair follicles.^17^ Previously, it was believed that if a wound extended deeper than the roots of the hair and sweat glands (the stumps of which act like a wound-edge epidermis), then these structures would not regenerate. However, this dogma is now in doubt because of new murine studies in which hair follicles are seen to arise *de novo* from apparently nonfollicular epidermis, in a Wnt-dependent manner that appears to recapitulate embryonic appendage formation.^18^

## Wound granulation tissue and dermis replacement

Many genes are also upregulated in the wound-edge fibroblasts. It was previously assumed that these cells were the sole source of the wound granulation tissue that became activated by exposure to various growth factor signals and are triggered to proliferate and migrate in synchrony with the advancing epidermis. Some of these cells transform into the contractile specialist cell, the myofibroblast, after exposure to transforming growth factor (TGF)-β and mechanical loading signals.^19^ But it now seems probable that at least a subset of fibroblasts within wound granulation tissue is not derived locally but rather originates from a bone-marrow-derived, mesenchymal stem cell (MSC) pool. By tracking fluorescent MSCs after intravenous injection into mice, several groups have reported a significant contribution by MSCs to the wound fibroblast population.^20^–^22^ A recent study showed that two populations of local stem cells exist within the dermis: one superficially, which is critical for hair development, and one deeper, forming the lower dermis, which is the likely source of early granulation-tissue fibroblasts.^23^

## Inflammation is good and bad during skin repair

Studies of repair in embryonic model organisms including mice, and also in human patients undergoing fetal surgery, have indicated that prior to the onset of a wound inflammatory response, immature tissues are capable of scar-free healing.^24^,^25^ These observations suggest that inflammation might be driving wound fibrosis. Indeed, mice lacking the family transcription factor PU.1, and thus not able to generate any leucocytic lineages, are nonetheless able to heal wounds effectively as neonates, and do so without subsequent scarring, unlike their wild-type littermates.^26^

There are now a plethora of mouse studies designed to test individually the function of most immune cell lineages in the wound repair process. For some lineages the jury is still out, but the current consensus is that early-recruited neutrophils largely deal with killing invasive microorganisms at the wound site,^27^ whereas macrophages are needed for clearing apoptotic neutrophils and orchestrating early wound closure events, and also emit signals that cause later scarring.^27^,^28^ By contrast, mast cells appear to play only fine-tuning roles during wound repair, because their genetic depletion leads to almost entirely normal healing.^29^,^30^ Other immune cell lineages are less well studied and may become involved in the repair process only if it becomes chronic. Currently very little is known about the role of adaptive immunity in the normal wound healing process, but one study of γδ T cells suggests that these may be vital for recognizing keratinocyte ‘damage’ signals and releasing key growth factors for epidermal migration.^31^

Inflammatory cell recruitment and activation are a consequence of many signals that occur at the wound site, and some of the earliest of these include factors released by degranulating platelets^32^ and by damage- and pathogen-associated molecular patterns, where cells are damaged and microbes gain access, respectively.^33^ All of these signals are potential therapeutic targets for modulating the initial wound inflammatory response in humans. A newly discovered and clinically relevant signalling pathway is triggered by wound mechanics; as a wound gapes open and then begins to contract, focal adhesion kinase/extracellular signal-regulated kinase leads to activation of chemokine ligand 2 release by wound fibroblasts, which, in turn, draws in a larger inflammatory response.^34^ Blocking any of these steps leads to reduced scarring in mice,^34^ supporting the theory that inflammation is the primary driver of scarring at the wound site. An alternative strategy for dampening the wound inflammatory response is to treat with known resolving factors, and this approach can also lead to reduced scarring.^35^ TGF-β1 is almost certainly one of the growth factors downstream of the wound inflammatory response, and knockdown of this signalling axis has been shown to reduce scarring.^36^ What remains unclear is precisely how inflammation-triggered molecular changes in wound fibroblasts, which include upregulation of osteopontin^37^ and other ‘fibrosis’ markers, subsequently lead to deposition and bundling of collagen fibres in ways that lead to pathological scarring.

## Wound angiogenesis and innervation

In the clinic, it is presumed that the considerable vascular sprouting that occurs during any adult tissue repair process must play a pivotal role in healing, and there is much clinical anecdote that cutaneous innervation is important also. Neither of these episodes has been extensively researched in the context of repair, but much is known about the development of vascular patterning during embryogenesis, where we know that endothelial cell sprouting is driven by vascular endothelial growth factor, and macrophages are important in these episodes.^38^–^40^ As for a role for nerves in the repair process, the amazing regenerative capacity of the axolotl may offer insight.^41^ Nerves turn out to be an intriguing contributor to the limb regenerative process whereby axon-ensheathing Schwann cells are known to release an early pulse of signals, including the secreted newt anterior gradient (nAG) protein (of which there are mammalian orthologues). The release of nAG from nerves appears to kick-start expression of nAG in the wound epithelium.^42^ Without this initial nerve signal, the wound stump heals but no limb grows back in newts. Very little is known about the role of nerves during skin healing, although studies in the chick embryo suggest a reciprocal positive association between nerves and wound repair.^43^

## New wound healing models: *Drosophila* and zebrafish offer translucency for live imaging, and considerably better genetic tractability

Much of what is known about the molecular and genetic aspects of skin wound healing has been gleaned from studies in mice, alongside some descriptive clinical observations. But the tissues of mouse and man are opaque and neither organism is particularly genetically tractable. These limitations have encouraged wound healing studies in *Drosophila*^44^ and zebrafish.^45^ Of course, flies and fish will not perfectly model human tissue healing, but their translucency offers opportunities for live imaging and their genetic tractability allows insights into fundamental and conserved tissue repair mechanisms that were not possible before.

In the *Drosophila* embryo, for example, movies of haemocytes (the fly equivalent of macrophages) that are mutant for each of the Rho-family small GTPases have revealed precise roles for these regulators of the cytoskeleton as fly macrophages undergo the wound inflammatory response.^46^ These molecular mechanisms can almost certainly be extrapolated to neutrophils and macrophages migrating to human wounds. Although the advancing wound epidermis is hidden beneath a scab in mouse wounds, the simpler fly epidermis can be live imaged, revealing dynamic cytoskeletal machineries, including lamellipodial and filopodial protrusions that enable fusion of epidermal wound edges together at the end of the healing process.^47^ Again, this is almost certainly revealing what we cannot see but is occurring in mouse and man.

Recent studies in *Drosophila* have shown that there are many active cell-shape changes and junctional alterations as epidermal cells jostle several rows back from the wound edge, and this will focus attention away from the front row of cells, which have been considered the only key players until now.^48^ Several genetic screens have been performed on embryo and larval *Drosophila* wound models to identify differentially expressed genes and mutants that suffer impaired healing;^49^,^50^ some of these are unique to flies, but others have highlighted conserved transcriptional activator pathways, including *wnt* and *Grh*,^51^ that have been shown to extrapolate to mammalian repair^52^,^53^ and may be good therapeutic targets.

Translucent zebrafish larvae offer a phylogenetic step up from *Drosophila*, with greater parallels to our own repair machinery. For example, rather than a single immune cell lineage, as in *Drosophila*, they have equivalents of all of our innate immune cells. Currently the most exciting insight from zebrafish studies of wound inflammation has been that reactive oxygen species like hydrogen peroxide can serve as immediate damage attractants to draw immune cells to wounds.^54^ Zebrafish are also beginning to offer clues to the endogenous mechanisms for resolution of inflammation. For example, neutrophils may be partly responsible for their own resolution by clearance of the attractants that first drew them to the wound.^55^

In addition to studies of inflammation, there are now new models of skin healing in adult zebrafish that reveal considerable parallels with mammalian wound repair, including similar, although faster, re-epithelialization, and transient scarring, all driven by conserved signalling pathways.^56^ This will provide further opportunities to use zebrafish for high-throughput, small-molecule drug screens, as an initial filter for testing potential therapeutics to improve healing in the clinic.

## Biology of chronic wound healing

**Figure 2:**
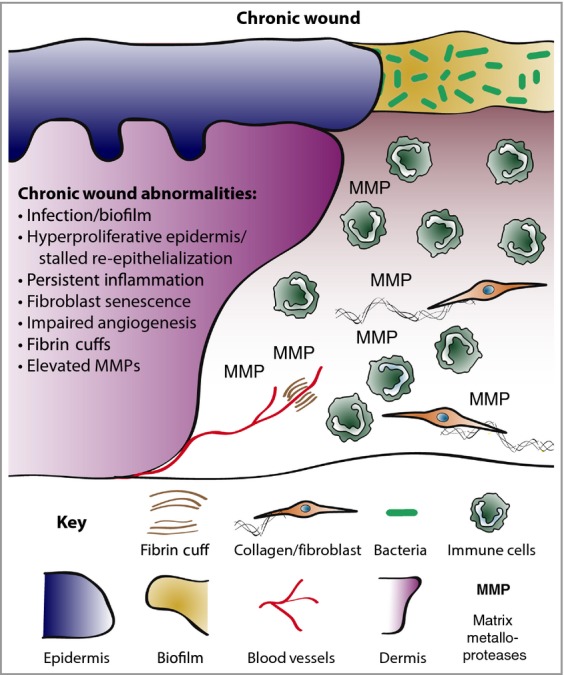
Chronic wound biology. Chronic wounds are often infected and exhibit a persistent aberrant inflammatory profile. Re-epithelialization stalls but wound keratinocytes are hyperproliferative. Granulation tissue is defective and does not nurture healing, in part due to elevated matrix metalloproteases (MMPs) and poor fibroblast infiltration. Neoangiogenesis is poor and fibrin cuffs restrict existing vessels, limiting the diffusion of oxygen through the wound, rendering the wound hypoxic.

Chronic wounds – diabetic foot ulcers, venous leg ulcers and pressure ulcers – do not adhere to the standard time course of cellular and molecular events that lead towards healing of a healthy acute wound (Fig.2). As an example, histological studies of chronic venous leg ulcers show a characteristic piled-up and hyperproliferative epidermal edge, abutting an ulcer base that is covered with exudate loaded with necrotic debris. Where there should be wound granulation tissue there are vessels surrounded by fibrin cuffs (presumed to be a response to venous hypertension), very little vessel sprouting and few, if any, myofibroblasts. There is generally a heavy inflammatory infiltrate, particularly of neutrophils, and these cells may be phenotypically different from their equivalents in a healing acute wound.^57^

Frequently, hyperpigmentation as a consequence of melanocyte recruitment can occur at the wound site, and persist even after a chronic wound has successfully healed. At a molecular level, it seems that the chronic wound edge keratinocytes express a gene signature reflecting partial proliferative activation, with several cell-cycle genes – including the cyclins – upregulated, but with suppression of checkpoint regulators and p53; this might explain the epidermal hyperproliferation at ulcer wound edges.^58^ The ulcer wound fibroblasts appear senescent, have diminished migratory capacity^59^ and appear unresponsive to growth factor signals,^60^ which is reflected in dramatically reduced levels of TGF-β receptors and downstream signalling cascade components from biopsies of nonhealing ulcers.^60^ An additional explanation for reduced growth factor signalling and responsiveness may be the increased levels of degrading MMPs in chronic vs. acute wound-tissue fluids.^61^ Potentially more informative for prognostic purposes than comparisons of chronic vs. acute wounds are comparisons of healing vs. nonhealing chronic wounds, and one such microarray study reveals major downregulation of the wound-associated keratin 16 and its heteropolymer partners keratins 6a and 6b in the nonhealing wound group.^62^

Chronic, persistent inflammation is a hallmark of most chronic wounds,^63^ whereas during acute healing, the normal pathway is for resolution of the inflammatory response. Of course, it is difficult to distinguish whether the continual open wound with exposure to microbes is causal of the chronic inflammation, or vice versa, or both. For some immune cell lineages in some chronic wound scenarios, more may be better; for example, increased numbers of Langerhans cells in the epidermis of diabetic foot ulcers have been shown to associate with better healing outcome.^64^ However, in general, a large influx and retention of innate immune cells into chronic wounds is likely to inhibit many repair processes. Even some of the useful functions of immune cells may be disrupted in chronic wounds, as it seems that their bactericidal and phagocytic activities may be reduced, in comparison with those in an acute wound setting.^65^ Perhaps as a result of the reduced phagocytic capacity of immune cells at the chronic wound, one consistent obstacle in the healing of many chronic wounds is a build-up of necrotic debris at the wound edge; as a consequence it is often clinical practice to debride the wound mechanically, to establish a ‘fresh new’ wound, which can help to restart the re-epithelialization process.^66^

With the growth of microbiome 16S ribosomal RNA sequencing opportunities, it is now possible to survey the full microbial flora of wounds, and early datasets are revealing some common genera between diabetic and venous leg ulcers, and significant differences also, whereas the microbial community across a sample of pressure ulcers appears to be the most variable.^67^,^68^ Almost certainly some of these pathogens, and even excessive numbers of some otherwise commensal species, might be key in modulating the efficiency of healing, either directly by their actions on keratinocytes or wound fibroblasts, or indirectly by modulating the inflammatory response. The next investigative steps will need to include a similar characterization of fungal and viral infections in chronic wounds. What is clear is that there will be a complex interplay between invading agents and the host immune system, and quite possibly the best prognostic signatures for indicating likely healing outcomes of chronic wounds will combine both microbiome and host transcriptome/metabolome components.

It is also important to note that many models of pathological wound healing in mice, while accurately mirroring some of the systemic causes of impaired healing (e.g. the obese and hyperglycaemic diabetic (*db*/*db*) mouse), seldom make allowance for other important associations, such as age and wound microbial load. There is a strong case to be made for improving such models by layering on some of these additional influencing factors so that data can be more usefully extrapolated to the clinic. This would certainly lead to development of more optimal models (as reviewed by Nunan *et al*.^69^).

## What causes wound scarring?

One of the mysteries in the field of tissue regeneration and repair is the heterogeneity among diverse organisms: some animals, including axolotls, can perfectly regenerate injured tissues and organs as complex as limbs, whereas others, like humans, replace damaged tissue with a connective tissue characterized by densely bundled orientated collagen fibrils called a scar. The degree of fibrosis after damage varies across organs and tissues and between individuals. In humans, perfect scar-free tissue repair has been described in fetal skin.^70^ Postnatal human liver, the haematopoietic system and, to a lesser degree, the gut and skin represent tissues that maintain the highest regenerative capacity.

**Figure 3:**
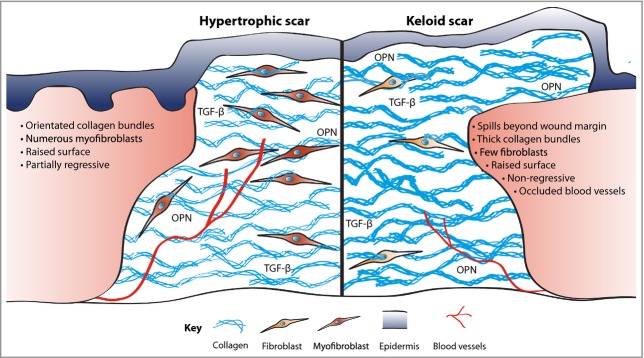
Excessive fibrosis. Scars, formed in part as a consequence of inflammatory signals, are comprised of collagen deposited in thick orientated bundles rather than the basket-weave-like fibrils found in normal dermis. Hypertrophic scars have excessive collagen deposition, leading to a raised surface that partially resolves over time. In contrast, keloid scars have thicker collagen bundles, extend beyond the original wound margin and rarely regress. Contractile myofibroblasts are prevalent in hypertrophic scarring but all but are absent in keloid tissue. Keloids can also be characterized by occluded blood vessels. OPN, osteopontin; TGF-β, transforming growth factor-β.

In human skin, two types of scarring following injury are distinguished: hypertrophic scars and keloids (Fig.3). Aesthetically disturbing hypertrophic scars develop after surgery or from other trauma, particularly burns. Keloids differ from hypertrophic scars in that they extend beyond the margins of the original tissue damage, and they do not regress spontaneously (hypertrophic scars generally partially regress within 6 months). Moreover, keloids tend to show a genetic predisposition, with particular association with darker-skinned populations.^71^ Keloids and hypertrophic scars can also be histologically distinguished by their different arrangement of collagen fibres, presence of α-smooth muscle actin-positive myofibroblasts, and extent of angiogenesis.^72^,^73^ Scarring can cause functional disability, for example if extended over a joint, or may cause patient discomfort and psychological stress.

Both hypertrophic scars and keloids are major therapeutic challenges for surgeons and dermatologists. Although multiple treatment regimens are practised, including silicone gel sheeting, pressure therapy, corticosteroids, cryotherapy, 5-fluorouracil, laser therapy and radiation, none of these is optimal and effective, and therapeutics based on molecular targets have not yet gone beyond clinical trials.^74^ Novel therapies for the treatment of cutaneous pathological scarring can potentially be extrapolated from clinical trials targeting fibrosis in other organs including lung, liver and kidney.^75^ Although tissue-specific features of fibrogenesis appear to exist, there is increasing appreciation of common pathways of fibrosis that are conserved among tissues, including TGF-β, connective tissue growth factor, interleukins 4 and 13, platelet-derived growth factor and osteopontin.^76^ Perhaps the closest to therapeutic treatments for blocking scarring in the skin have been the various approaches used to modulate TGF-β1/2 signalling immediately following wounding, either by blocking receptor activation or by addition of the competing ligand TGF-β3 to the wound.^36^ Approaches such as these will eventually lead to scar-blocking therapeutics in the clinic.

## Important future directions for wound healing research

In order to aid advancement of wound healing research in directions that will lead to benefits in the clinic, we need a good dialogue between clinicians and basic scientists. There follows just a few of the key unmet needs that might be worthy of research and provide clues as to prognostics and therapeutics for chronic wounds and for scarring.

### When during the normal cycle of repair does a chronic wound stall?

We know that almost all chronic wounds begin as a small cut or abrasion, and almost certainly begin the repair process as a normal acute wound. At some stage they stall, but of course this is likely to be some days or weeks or even months before the patient presents at the clinic. Unfortunately, we have little understanding of the time or stage in the normal cycle when stalling happens, and this may be crucial in developing therapeutics to reverse the failed process. There is a clear correlation between chronic wound duration and healing efficacy,^77^ but more precise biomarkers to indicate key stages in the normal repair process would certainly be useful here and might also serve as prognostic indicators.

### Is there a microbe/host transcriptome signature that predicts healing outcome, and can the immune response in chronic wounds be reprogrammed to be better at killing wound pathogens?

It is now well understood that innate immune cells exhibit various phenotypes or activation states that can be either very antimicrobial or more dedicated towards nurturing of repairing tissue by their release of growth factors and cytokines. Learning how to manipulate or reprogramme the inflammatory response so it is most effective at staving off infection, and then able to switch to repair mode, and finally to resolve in a timely fashion to avoid the chronic inflammatory phenotype so common in persistent chronic wounds, would provide superb therapeutic tools. If we knew which microbe combinations and what host response led to the most stubborn non-healing wounds then we would have very useful prognostic indicators to guide which wounds need what treatment.

### Can hypertrophic excessive scarring be dampened or reversed without affecting the rate and quality of skin wound healing?

It is generally believed that aspects of the acute wound inflammatory response drive scar formation at the time when skin wound healing is occurring – but can inflammation or its downstream consequences be modulated in ways that allow efficient healing but reduce scarring? One opportunity here might be to utilize the body's own inflammation resolution signals to drive early ‘shutdown’ of the inflammatory response.^35^ A better understanding of precisely how wound fibroblasts alter their behaviour, after receiving ‘fibrosis’ signals from inflammatory cells, and subsequently deposit collagen in pathological ways would also offer insight into antifibrotic therapeutic targets.

### Is the molecular basis for keloid scarring similar to that for hypertrophic scarring?

If inflammation causes hypertrophic scarring, then does even more inflammation cause the ‘overflowing’ keloid scar? Or is keloid scarring due to an inability of wound fibroblasts to respond to wound ‘stop’ signals and, if so, what are these? There are no animal models of keloid scarring, but there are opportunities for genome-wide association studies to identify genes that might predispose to keloid scarring, and these might also lead us towards more generic antiscarring therapies if the mechanisms of hypertrophic and keloid scarring are at all related.

Much is known about the cellular and molecular basis of normal skin healing, but there are still avenues of research left to unravel that will guide us towards better prognostic indicators and better therapeutics for the various skin wound healing pathologies reviewed above.
